# Improvement of Chronic Neck Pain After Posterior Atlantoaxial Surgical Fusion via Multimodal Chiropractic Care: A Case Report

**DOI:** 10.7759/cureus.34630

**Published:** 2023-02-04

**Authors:** Eric Chun-Pu Chu, Robert J Trager, Cliff Tao

**Affiliations:** 1 Department of Chiropractic and Physiotherapy, New York Chiropractic and Physiotherapy Centre, Kowloon, HKG; 2 Chiropractic, Connor Whole Health, University Hospitals Cleveland Medical Center, Cleveland, USA; 3 Radiology, Private Practice of Chiropractic Radiology, Irvine, USA

**Keywords:** cervical vertebrae, atlantoaxial fusion, neck pain, spinal manipulation, chiropractic

## Abstract

There is a lack of research regarding the effectiveness and safety of manual therapies, including spinal manipulative therapy (SMT), for patients with previous cervical spine surgery. A 66-year-old, otherwise healthy, woman who underwent C1/2 posterior surgical fusion for rotatory instability during adolescence presented to a chiropractor with a six-month history of progressive worsening of chronic neck pain and headaches despite acetaminophen, tramadol, and physical therapy. Upon examination, the chiropractor noted postural changes, limited cervical range of motion, and muscle hypertonicity. Computed tomography revealed a successful C1/2 fusion, and degenerative findings at C0/1, C2/3, C3/4, and C5/6, without cord compression. As the patient had no neurologic deficits or myelopathy and tolerated spinal mobilization well, the chiropractor applied cervical SMT, along with soft tissue manipulation, ultrasound therapy, mechanical traction, and thoracic SMT. The patient’s pain was reduced to a mild level and the range of motion improved over three weeks of treatment. Benefits were maintained over a three-month follow-up as treatments were spaced apart. Despite the apparent success in the current case, evidence for manual therapies and SMT in patients with cervical spine surgery remains limited, and these therapies should be used with caution on an individual patient basis. Further research is needed to examine the safety of manual therapies and SMT in patients following cervical spine surgery and determine predictors of treatment response.

## Introduction

While evidence and clinical practice guidelines support the use of manual therapies including soft tissue manipulation and spinal manipulative therapy (SMT) for neck pain [1,2], there is limited research on the safety and use of these therapies in patients who have previously undergone cervical spine surgery [3]. According to a scoping review in 2022, there were only three published cases in which SMT had been used for a patient with a previous cervical spine surgery [3]. Accordingly, there is limited information to inform clinicians’ use of SMT or other manual therapies in such patients [3]. To our knowledge, no previous research has described the use of manual therapies or SMT in a patient with surgical fusion at C1/2.

Atlantoaxial (e.g., C1/2) surgical fusion may be indicated in patients with joint instability at this level, such as those with congenital bony or ligamentous abnormalities, trauma, inflammatory conditions, or neoplasms [4-6]. Atlantoaxial instability is a rare but serious condition that may lead to spinal cord compression [7,8]. A related condition, atlantoaxial rotatory subluxation, involves rotational displacement of the C1/2 facet joints and predominantly affects pediatric patients [9]. Surgical approaches to C1/2 instability traditionally used cerclage wire and onlay bone graft [10,11]. However, newer methods involving screw fixation have become more common due to having a higher rate of fusion (i.e., 99% versus 83%) [5].

Patients may have persistent, recurrent, or worsening symptoms after cervical spine surgery, even when the surgery is initially successful [12]. For example, patients may experience a recurrence of their initial problem or develop a new condition altogether, such as disc herniation [12]. Adjacent segment disease (ASD) is one potential cause of recurrent symptoms and describes accelerated degenerative changes in the joints neighboring a surgical spinal fusion [13]. ASD is thought to relate to biomechanical, surgical, or patient-related variables such as younger age and greater body mass index [13].

While ASD generally affects 28% of patients after surgical cervical fusion, its prevalence, risk factors, and clinical features in patients who have undergone C1/2 fusion have not been studied in-depth [14]. Regardless, authors suspect that surgical atlantoaxial fusion may contribute to ASD by increasing stresses and motion demands on the C2-7 articulations [15,16]. One study found that the angle of C1/2 fusion affected the cervical sagittal alignment in a compensatory fashion, with the angle of C1/2 inversely correlated to the C2-7 angle [16]. Another study found that experimental C1/2 fusion in cadavers increased mid-cervical intradiscal pressure during cervical flexion, yet this effect depended on the angle of C1/2 fusion [15].

Chiropractors are portal of entry providers that commonly manage spinal complaints such as back and neck pain [17]. Although chiropractors often use SMT as a treatment, there is limited research regarding the utility and safety of this therapy in patients who have undergone cervical spine surgery. Given patients with surgical cervical fusions often have persistent or recurrent neck pain and may seek chiropractic care, we present a case of an older woman with chronic neck pain and C1/2 posterior fusion who improved with multimodal chiropractic treatments.

## Case presentation

Patient information

A 66-year-old, otherwise healthy, woman with a history of C1/2 posterior surgical fusion presented to a chiropractor with incapacitating neck pain, spasm, head tilt, and right occipital and periorbital headaches which had progressively worsened over the past six months and failed to improve with acetaminophen, tramadol, and physical therapy. She reported her mean pain intensity was 7/10 and noted that her symptoms were more severe in the morning and were partially alleviated by stretching her neck from side to side. She denied having any bowel or bladder symptoms or any family history of neurological or spinal disorders or cancer. Her World Health Organization Quality of Life (WHOQOL) score was 76%. Until recently, she was relatively active walking regularly. She was retired from a secretarial desk job, was a non-smoker, and social drinker.

In 1970, at age 14, the patient suddenly developed severe neck pain, torticollis, and headaches which were aggravated by sneezing and neck movements and alleviated by holding her head with her hand, and after imaging was diagnosed with C1/2 “rotatory instability.” To her knowledge, there was no precipitating factor such as trauma or infection. She was treated using 24-hour halo traction for six months. As her symptoms remained the same, she then was placed in a Minerva cast (i.e., from the head to the iliac crests, leaving the face exposed) for an additional six months, yet this did not resolve the symptoms. At age 16, she underwent posterior surgical fusion of C1/2 which was performed using wire and an on-lay bone graft from the iliac crest placed between C1 and C2. Post-operatively, she had severe bilateral lower extremity weakness and was unable to walk, requiring the use of a wheelchair for three months. She also wore a cervical orthosis during this time. She made a nearly full recovery by six months after surgery without neck pain or headache. Her only residual symptom was a sensation of disconnection of her lower torso when sleeping.

In 2015, at age 60, she tripped while walking and fell, and progressively developed cervicalgia, occipital headaches, and involuntary, jerking neck movements in her anterior neck muscles, which occurred every few hours according to the patient’s description. The patient visited a neurologist, and after undergoing radiographs was diagnosed with degenerative changes in the spine and prescribed acetaminophen.

Six months prior to presenting to the chiropractor, the patient visited the emergency department due to worsening neck pain and headaches which were severe at this point. The patient underwent cervical radiographs which were reported to show degenerative changes. She also underwent laboratory studies including a complete blood count, renal profile, electrolyte panel, calcium, magnesium, C-reactive protein, liver, and thyroid testing, all of which were unremarkable. She subsequently visited her primary care provider and was prescribed tramadol and referred for physical therapy where she performed upper extremity strengthening exercises and neck stretches. As her symptoms failed to improve, she presented to a chiropractor for additional options.

Clinical findings

**Figure 1:**
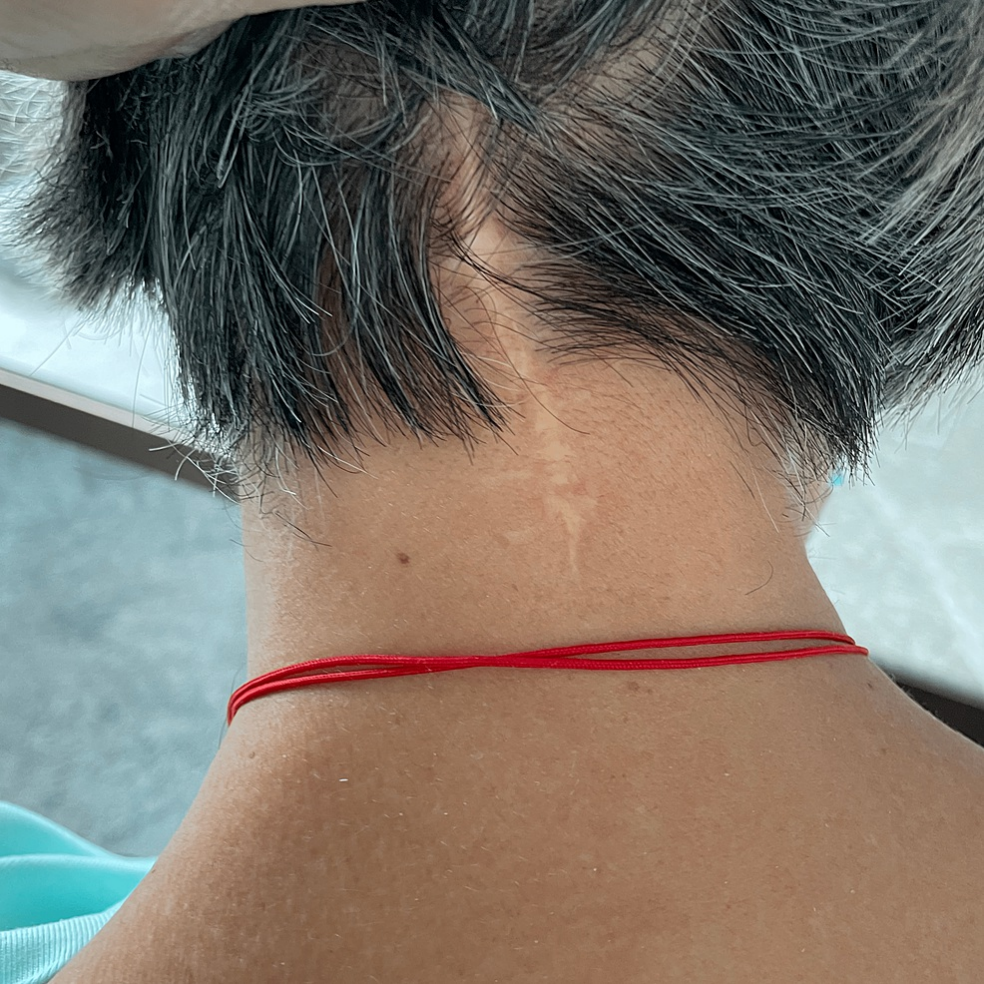
Observation of the patient’s posterior neck A well-healed surgical scar was evident from the occipital region to the mid-cervical spine. Note that the patient is holding her hair which slightly affects the position of her neck.

Upon physical examination by the chiropractor in 2022, the patient’s neck was observed to be slightly rotated to the left side and laterally flexed to the right at rest. Well-healed surgical scars were visible at the upper cervical (Figure 1) and left iliac crest region. The patient was well-oriented and attentive, displayed a normal gait, had no tremor or bradykinesia, and had normal coordination, a normal cranial nerve examination, and 2+ muscle stretch reflexes diffusely, without pathological reflexes. The patient’s active cervical range of motion was limited to 30° of flexion and extension, and 20° of lateral flexion, and 10° of rotation bilaterally. A motor examination revealed 4/5 strength of her right deltoid (Medical Research Council scale). Spinal palpation identified motion restriction at C5/6 and T5/6 spinal levels and muscular hypertonicity of the suboccipital muscles bilaterally.

**Figure 2:**
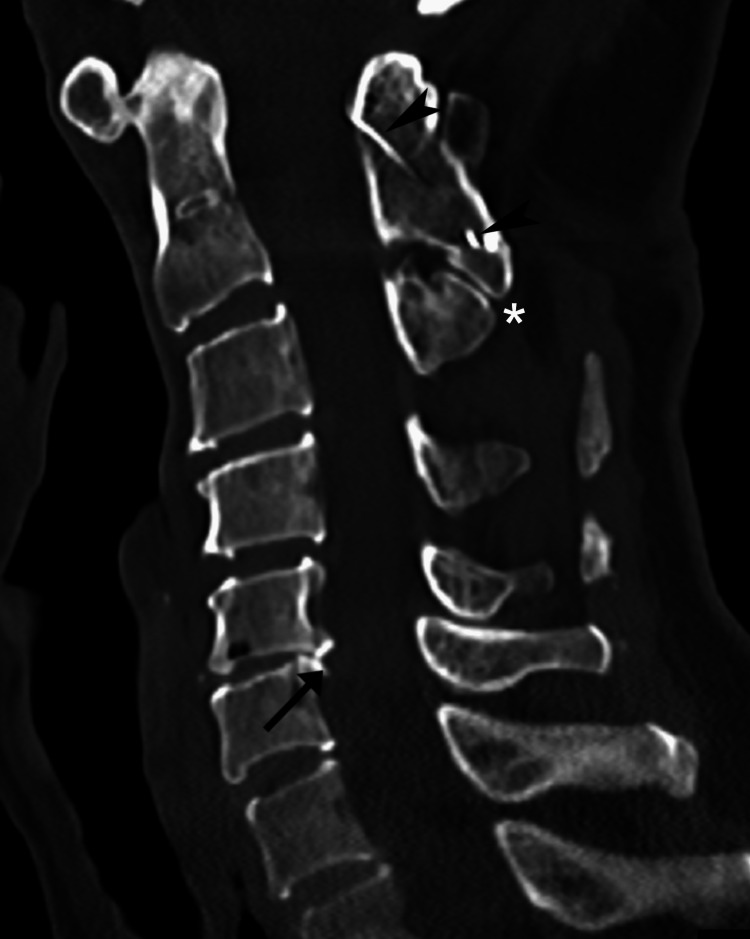
Cervical spine computed tomography, mid-sagittal section Cerclage wire (arrowheads) is evident from her previous procedure which was placed from the C1 posterior arch to the C2 spinous process with interposed autograft bone. Mild disc herniations are evident at C3/4 and C4/5, while the C5/6 disc space is severely narrowed and posteriorly has a disc-osteophyte complex (arrow), which causes mild narrowing of the spinal canal at this level to 8 millimeters in anteroposterior diameter and mild osteophytic encroachment of the left C5/6 neuroforamina (not shown). Interspinous narrowing and pseudoarticulation is evident between the C2 and C3 spinous processes (*). A slight focal kyphosis from C1 to C4 is also evident.

**Figure 3:**
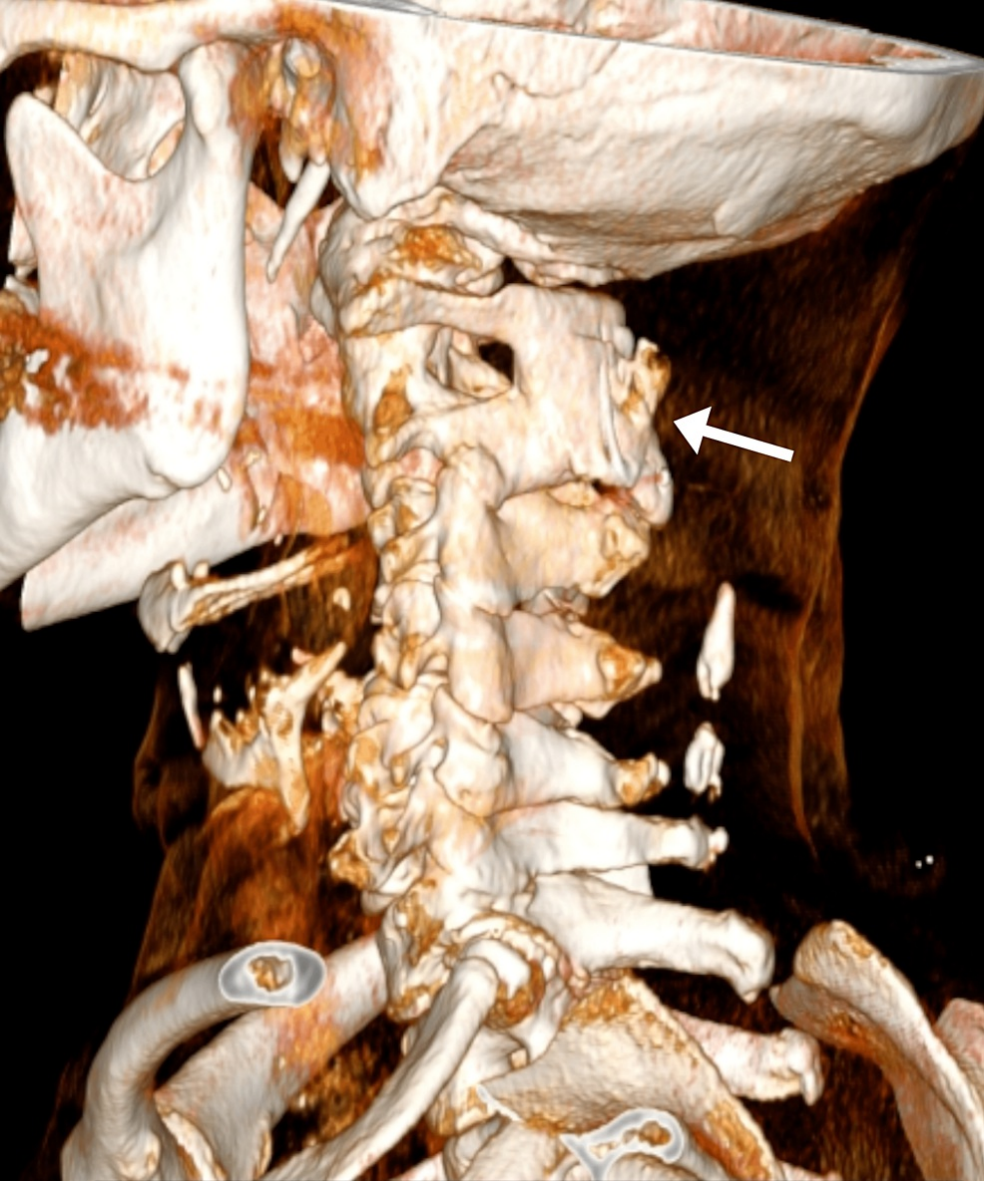
Three-dimensional reconstruction of the cervical computed tomography Successful posterior bony fusion between C1 and C2 is evident along with the interposed cerclage wire (arrow), which is not as easily appreciated in the two-dimensional views.

**Figure 4:**
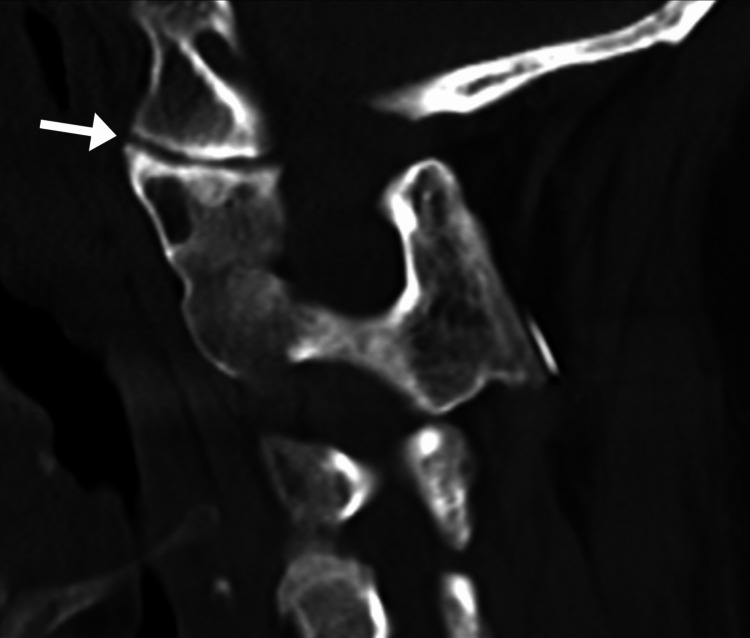
Sagittal computed tomography section through the left atlanto-occipital joint The joint is narrowed (arrow), consistent with mild degenerative changes (osteoarthritis).

The chiropractor’s working diagnosis was ASD, however myofascial pain and cervical dystonia were also considered. Given the patient’s progressive worsening, lack of response to previous conservative care, and history of cervical surgical fusion, the chiropractor recommended computed tomography (CT) to evaluate the spine prior to treatment. The chiropractor opted to avoid MRI as a precaution considering it was unclear if ferromagnetic materials had been used. The patient agreed to undergo CT which was performed that week and revealed a successful posterior cervical fusion at C1/2, pseudoarticulation between the spinous processes of C2 and C3, slight focal kyphosis, disc herniations at C3/4, C4/5, and a disc-osteophyte complex at C5/6 causing mild canal stenosis (Figures 2-3). Mild cord indentation was noted without any obvious cord compression. Also noted were mild degenerative changes at the left atlanto-occipital joint (i.e., C0/C1; Figure 4)

The chiropractor considered the patient’s symptoms to relate to the degenerative findings at C0/C1, C2/3, C3/4, and C5/6. While the degenerative changes at C0/C1 and C2/3 could be considered a type of ASD, the changes at C3/4 and C5/6 could represent post-surgical subaxial segment degeneration [15,16]. In addition, the chiropractor considered myofascial pain to contribute to the patient’s symptoms given her abnormal posture and muscle hypertonicity. Imaging findings did not reveal any significant foraminal or central canal stenosis, which was consistent with the physical examination and was not suggestive of radiculopathy or myelopathy. Although the CT revealed degenerative findings, the chiropractor considered there to be no absolute contraindications to manual therapies given the lack of severe neurologic deficits, lack of spinal cord compression, and success of the previous C1/2 fusion.

**Figure 5:**
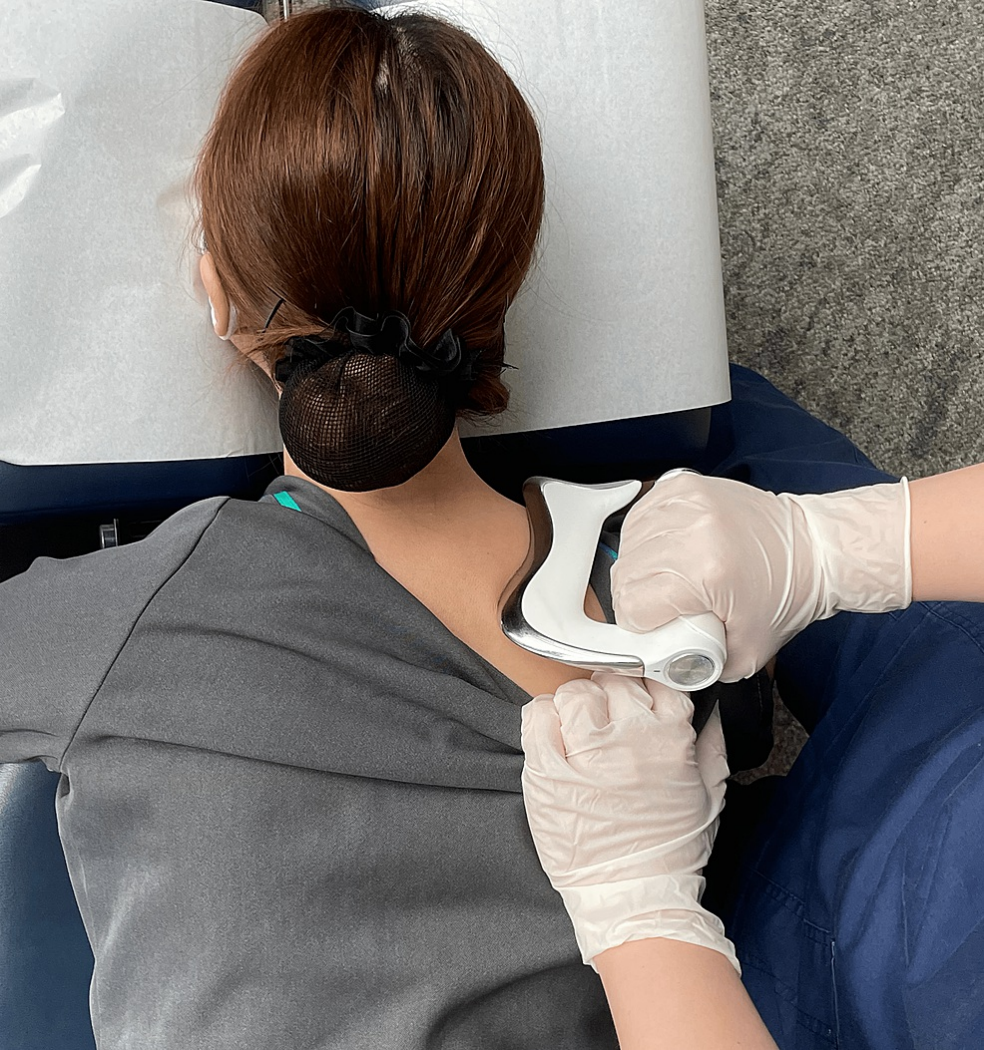
Demonstration of instrument-assisted soft tissue manipulation The chiropractor applies a thin layer of emollient to the cervical-thoracic region and gently strokes the massage tool (Strig, Korea) along the upper trapezius.

**Figure 6:**
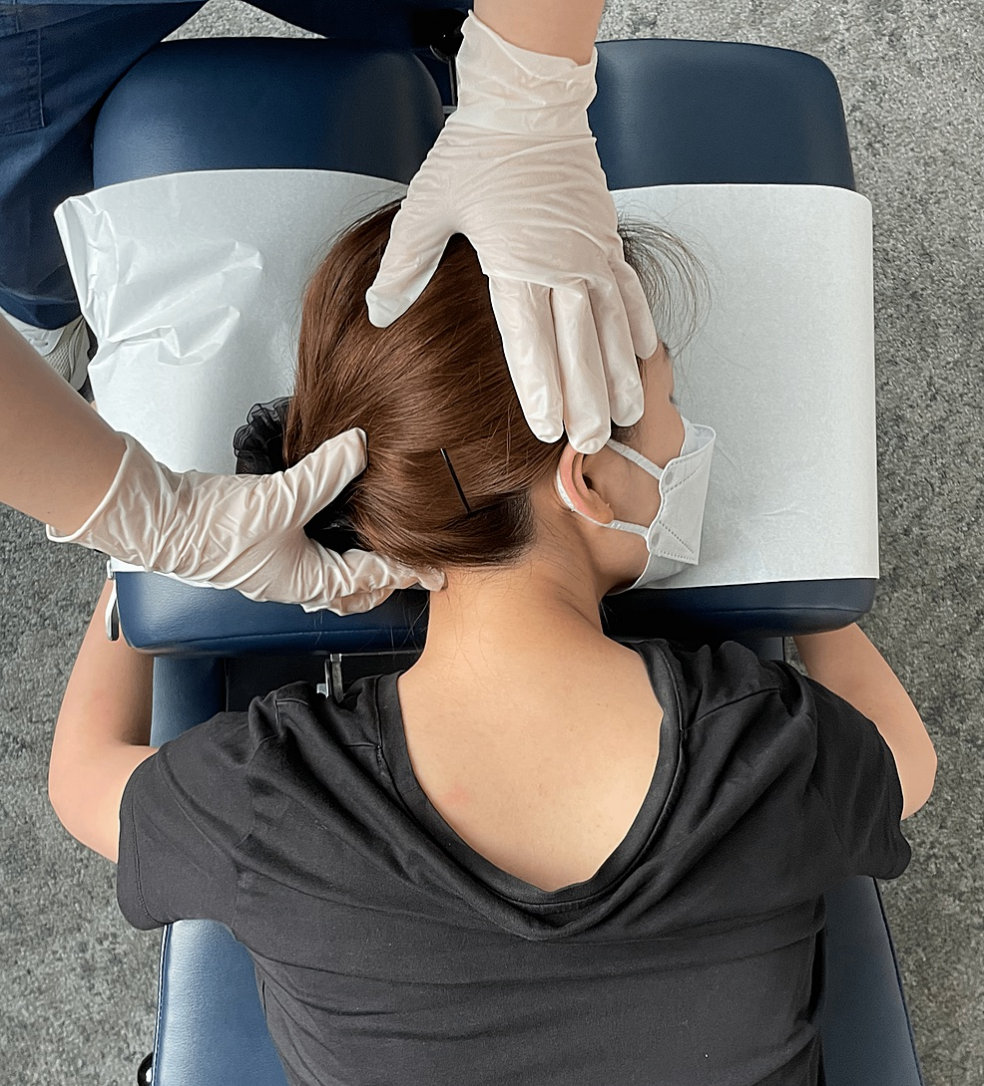
Demonstration of prone cervical mobilization The chiropractor stabilizes the patient's head against the headrest and provides gentle repetitive posterior to anterior pressure to the C5/6 articulations.

**Figure 7:**
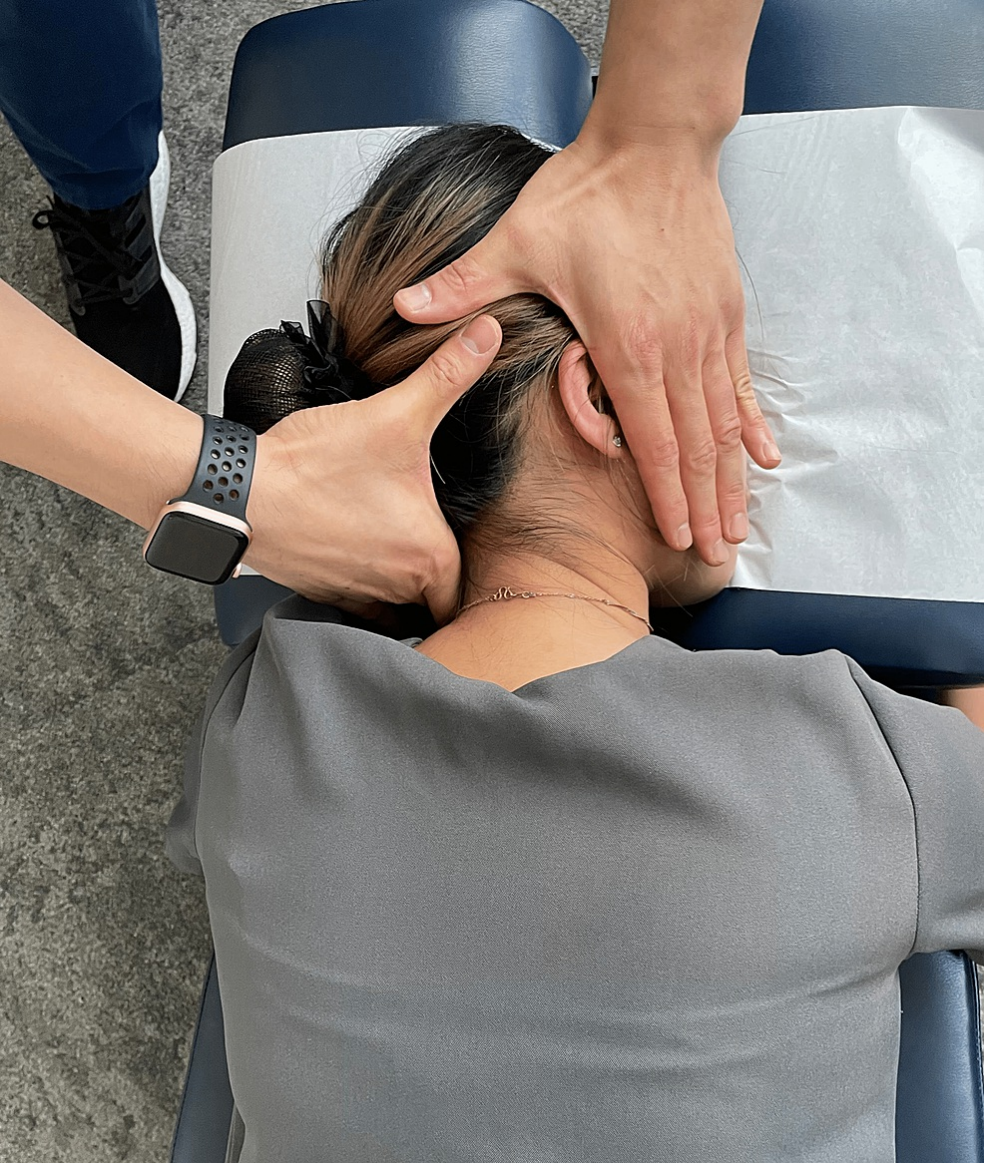
Demonstration of prone cervical manipulation The chiropractor stabilizes the patient's head against the headrest and provides a high-velocity, low-amplitude thrust directed posterior to anterior and lateral to medial at the C5/6 articulations using the 2nd digit.

The chiropractor recommended a trial of multimodal chiropractic care at a frequency of three visits per week for three weeks, to which the patient consented. The chiropractor applied instrument-assisted soft tissue manipulation using a massage tool (Strig, Korea) to the cervical suboccipital muscles, erector spinae, and upper trapezius bilaterally to reduce hypertonicity and alleviate pain (Figure 5). Therapeutic ultrasound was also applied to the posterior cervical musculature for eight minutes per visit. The chiropractor initially performed spinal mobilizations to the C5/6 segments (Figure 6). As the patient tolerated mobilizations well, the chiropractor then applied high-velocity, low-amplitude SMT at C5/6 (Figure 7). Thoracic SMT was also performed at T5/6 with the patient prone.

**Figure 8:**
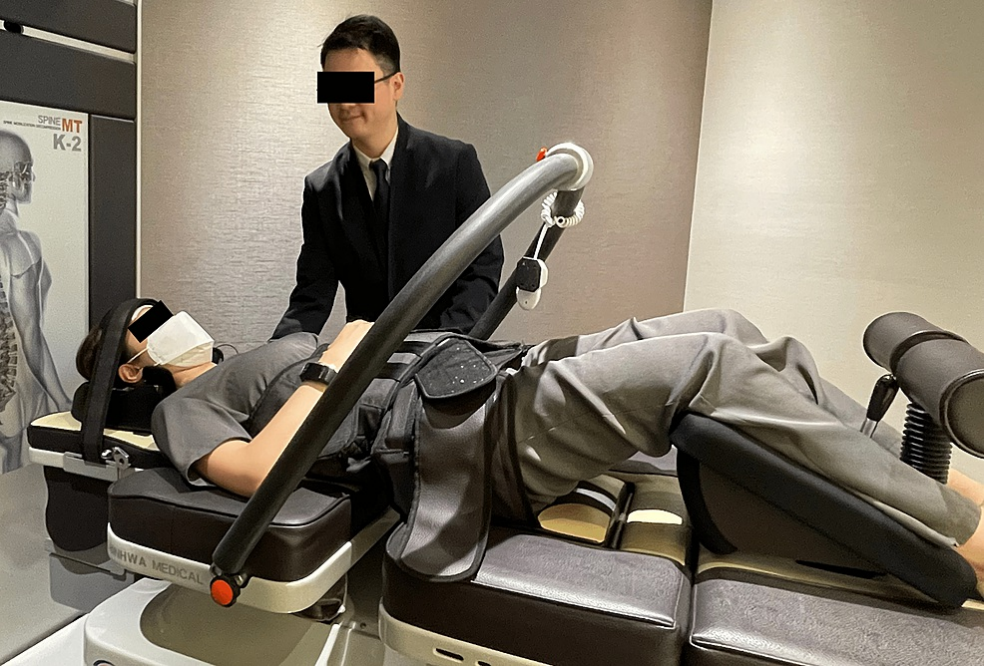
Mechanical cervical spine traction demonstration The patient lies supine on the table (Spine MT, Shinhwa Medical, Korea) with the knees gently flexed and the pelvis and head stabilized via straps. A traction force equaling 10% of the patient’s body weight is applied for 20 minutes.

After the first week of treatment, the patient’s mean pain severity reduced from 7/10 to 3/10. In addition, she reported that the abnormal sensation in her lower torso had completely resolved. During the second week of care, the chiropractor added mechanical cervical traction (Spine MT, Shinhwa Medical, Korea; Figure 8) to decompress the degenerative C5/6 disc, in addition to the soft tissue and spinal manipulation. Traction utilized a traction force of 10% of the patient’s body weight and was performed for 20-minute sessions at each visit. After three weeks of treatment, the patient reported complete resolution of her headaches, near complete resolution of her neck pain to a mild level (i.e., 1-3/10), and her WHOQOL score increased to 90%. Upon re-examination, her active cervical spine range of motion improved to 60° of flexion, 30° of extension, 20°of lateral flexion, and 30° of rotation to both sides.

Due to ongoing intermittent mild neck pain, the patient continued to follow-up with the chiropractor twice per week for the following two months. She subsequently presented for care once per week for the following month. She noted that the jerking motions in her neck resolved, however, her husband remarked that he still noticed a mild motion. At re-evaluation three months after her initial chiropractic visit, the patient’s WHOQOL score improved to 92% and her cervical range of motion maintained the same level of improvement compared to her prior examination performed after three weeks of chiropractic care. The patient provided written consent for the publication of this case report and any accompanying images.

## Discussion

This case report describes an older woman with a posterior C1/2 fusion and chronic, progressively worsening neck pain that failed to respond to medications and physical therapy, who improved with multimodal chiropractic therapies. The patient’s symptoms appeared to relate to degenerative changes at C0/1, C2/3, C4/5, and C5/6 rather than the site of original surgery (C1/2). These changes could have been precipitated by increased biomechanical stresses in the adjacent segments (i.e., ASD) and subaxial spine related to the C1/2 fusion, consistent with prior research on this topic [15,16]. According to a recent study, the average active cervical spine rotation is 74 ± 8°, with rotation at C1/2 accounting for approximately 50% of this motion [18]. Accordingly, cervical rotation would be expected to be significantly limited after C1/2 fusion, as in the current case. While some range of motion was regained with treatment (e.g., rotation 10° to 30°, flexion 30° to 60°), it was still expectedly limited.

We are aware of two similar cases in which SMT benefited a patient with previous cervical surgical spinal fusion. In one such case, a 52-year-old man with neck pain and a history of anterior discectomy and fusion at C5/6 was responded positively to instrument-assisted cervical spinal manipulation [19]. In another similar case, a 70-year-old man with cervicothoracic pain and a history of anterior cervical discectomy and fusion from C3-7 responded positively to soft tissue manipulation and high-velocity, low-amplitude spinal manipulation at the cervicothoracic junction [20].

In contrast, another case reported an adverse event in relation to SMT provided for a patient with a previous cervical surgical fusion. In this case, a 59-year-old man presented to a chiropractor with chronic neck pain and had a history of anterior discectomy and fusion at C6/7 [21]. After undergoing SMT, he developed motor deficits and gait abnormality, and required additional surgery for at disc herniation at C5/6 [21]. Unfortunately, this case did not provide details regarding the patient’s symptoms prior to receiving SMT (e.g., presence/absence of sensorimotor deficits) and further details related to the chiropractic treatments rendered (e.g., method of SMT).

The current case was unique in that the patient’s surgery was confined to the upper cervical segments rather than the subaxial segments as in the previous cases. Accordingly, several mid-to-lower cervical motion segments remained which were potential targets for SMT. Further, the current patient had no apparent neurological deficits which contrasts the two previous cases reporting these details [19,20]. It is possible that the location of the surgery and lack of deficits made the current patient a good candidate for SMT, however, this is unclear given the lack of research on this topic.

The therapies used in the current case have several mechanisms that may account for the reduction in the patient’s pain and improvement in mobility. SMT is theorized to have anti-nociceptive (i.e., pain-reducing) effects, the efficacy of which may depend on patients’ expectations [22], and may improve intersegmental motion [23]. Instrument-assisted soft tissue manipulation has been likewise shown to reduce pain and improve mobility [24]. Additionally, thoracic spinal manipulation, as used in the current case, has been found to be effective in alleviating cervical spine pain [25,26].

The safety and effectiveness of manual therapies for patients with previous cervical spine surgery remain unclear given the limited research on the topic. Therefore, we suggest that these treatments should be used cautiously on a case-by-case basis. Certain clinical variables such as patients’ age, the length of time since surgery, the presence of neurologic deficits, the type of surgery, the presence of surgical implants, or the number of spinal segments involved in the surgery could be relevant toward influencing a providers’ decision to use manual therapy in these patients, or the type of manual therapy used [27-30].

In addition, there is a lack of consensus in the chiropractic profession regarding the optimal treatment approach for patients with previous cervical spine surgery. One survey of Asia-Pacific chiropractors (n=241) asked if respondents would use cervical thrust SMT for a hypothetical 45-year-old female with localized neck pain and previous C5/6 fusion [30]. About half (53%) of respondents indicated that they would not, while 39% indicated they would, and 8% were unsure [30]. For the same hypothetical patient, except with a multilevel fusion from C3-6, a greater percentage of respondents indicated (63%) that they would not use cervical thrust SMT [30]. However, this survey did not examine respondents’ perspectives on patients with C1/2 fusion, as in the current case. Further research is needed to understand how chiropractors manage patients with neck pain and prior cervical spine surgery and which treatment methods are safest and most effective.

The current case has certain limitations. Given that a combination of therapies was used, it is not clear which therapies were most effective. As the patient likely had non-ferromagnetic cerclage wires in the upper cervical spine, it is possible that magnetic resonance imaging could be safely performed to further evaluate the cervical spine. However, further imaging could not be justified as the patient improved with care and had no signs of myelopathy or other red flags. Her previous radiographic images could not be obtained upon request, and it was unclear how she developed atlantoaxial rotatory instability as an adolescent. Flexion-extension radiographs could have been obtained upon presentation to the chiropractor to assess for spinal instability. However, the provider deemed instability to be unlikely as the patient had a stiff neck with limited mobility and did not demonstrate any catching, clicking, or clunking with movement. Instability was also unlikely given that the CT did not reveal any spondylolisthesis [31]. Degenerative changes are common in the cervical spine in older patients [32], and the disc displacements in the mid-to-lower cervical region could have developed irrespective of the C1/2 surgical fusion. Considering the patient remains active in care, it is unclear if her symptoms would return if chiropractic care was terminated.

Further research is necessary to examine the effectiveness and safety of manual therapies in patients with previous cervical spine surgery. Considering these patient presentations may be uncommon, such research may be most feasible to conduct via additional case reports or series, or chart review studies. It is possible that these studies may elucidate which patients would best respond to manual therapies, and which type of manual therapies are most appropriate. We also encourage clinicians to publish any cases of adverse events related to manual therapies in such patients, which could likewise be informative.

## Conclusions

This case presents an older woman with chronic progressively worsening neck pain and headache and a history of posterior surgical fusion of C1/2 who improved with multimodal chiropractic therapies including SMT. Clinicians should be cautious in using manual therapies in patients with cervical fusion yet may do so on a case-by-case basis after considering the patient’s clinical features and type and location of surgery. Further research is needed to examine the safety of these therapies and identify which patients may respond best.
